# Establishment and Validation of a Non-Radioactive Method for *In Vitro* Transcription Assay Using Primer Extension and Quantitative Real Time PCR

**DOI:** 10.1371/journal.pone.0135317

**Published:** 2015-08-07

**Authors:** Juan Wang, Shasha Zhao, Ying Zhou, Yun Wei, Wensheng Deng

**Affiliations:** Institute of Biology and Medicine, Wuhan University of Science and Technology, Huangjiahu Campus, Wuhan, 430065, Hubei, China; University of Pittsburgh School of Medicine, UNITED STATES

## Abstract

Primer extension-dependent *in vitro* transcription assay is one of the most important approaches in the research field of gene transcription. However, conventional *in vitro* transcription assays incorporates radioactive isotopes that cause environmental and health concerns and restricts its scope of application. Here we report a novel non-radioactive method for *in vitro* transcription analysis by combining primer extension with quantitative real time PCR (qPCR). We show that the DNA template within the transcription system can be effectively eliminated to a very low level by our specially designed approach, and that the primers uniquely designed for primer extension and qPCR can specifically recognize the RNA transcripts. Quantitative PCR data demonstrate that the novel method has successfully been applied to *in vitro* transcription analyses using the adenovirus E4 and major late promoters. Furthermore, we show that the TFIIB recognition element inhibits transcription of TATA-less promoters using both conventional and nonradioactive in vitro transcription assays. Our method will benefit the laboratories that need to perform *in vitro* transcription but either lack of or choose to avoid radioactive facilities.

## Introduction

Primer extension-dependent *in vitro* transcription assay is one of the most widely used approaches in the research field of gene transcription [1–4], particularly useful in 1) analyzing the regulatory role of the gene promoter and promoter elements in transcription, 2) analysis of the mechanisms of transcriptional activation/repression and 3) the effect of transcription factor-promoter interaction on transcription [5–7]. Although the method has been used in transcription analysis for decades and formally published as a laboratory protocol [8],the conventional primer extension-dependent *in vitro* transcription assay requires a radioactive-labeled primer, followed by gel electrophoresis, gel drying and autoradiography, which can potentially restrict its application, and particularly hinders laboratories that do not have access to facilitiesfor handling radioactive substances.

Quantitative real time PCR is a very powerful tool that has been widely used in modern molecular biology including gene expression analysis, pathogen detection, gene contamination detection and clinical diagnosis due to its sensitivity and accuracy [9–12]. It has been described that the efficiency of the *in vitro* transcription assay is surprisingly low [13, 14], and thus we hypothesized that quantitative PCR (qPCR) could be alternative method to detect the products of transcription *in vitro*. In our previous study, we showed that the TFIIB recognition element (BRE) can inhibit the transcription activity of a core promoter containing a TATA box [6], however, whether BRE represses the activity of the TATA-less promoters remains unknown. In this study, we have established a novel non-radioactive method for cell-free *in vitro* transcription analysis in combination with DNA template elimination, primer extension and qPCR using specially designed primers. Our data demonstrate that the method can be applied to several types of *in vitro* transcription analyses and that the BRE inhibits the transcription activity of TATA-less promoters.

## Materials and Methods

### Plasmids, proteins and reagents

Adenovirus E4 core promoter (nucleotides -51 to +12, termed E4wt), Adenovirus major late promoter (nucleotides -50 to +22, termed MLwt) and theirmutant derivativescontaining a defective TFIIB recognition element (BRE) were cloned into the reporter vector pGL3 basic (Promega). For E4 mutant derivatives, BRE consensus bases downstream of the TATA box were mutated into TFIIB-hatred bases as described before (termed E4mBRE) [5]; whereas AdML mutant derivatives, BRE consensus bases both upstream and downstream of the TATA box were mutated into TFIIB-hatred bases as described previously (termed AdMLmBRE) [6]. The promoters for AdMLmutant derivatives with a defectiveTATA element (termed BRE-mT and mBRE-mT) were also cloned into pGL3 basic, in which the TATA was changed into the CGAT, the ADML mBRE was obtained by the mutation as described above. Wild type TFIIB, TFIIB mutant (G13Q:R154A) and Gal4-AH were prepared and purified as described previously [5]. HeLa nuclear extract was purchased from Computer Cell Center Co.; RNA extraction reagent Trizol from Life Technology, Real time PCR reagent IQ SYBR Green from Bio-Rad. All other reagents were purchased from Thermo Scientific.

### Cell-free in vitro transcription

Conventional *In vitro* transcription was performed in a cell-free system as described previously [5], however, transcription *in vitro* for nonradioactive method was slightly modified for the study. Briefly, the transcription reaction system contains 25 μL nuclear extract, 3 μL 100 mM MgCl_2_, 200 ng DNA template, 10 μLddH_2_O and 10 ng Gal4-AH activator (added only for activated transcription). The reaction was mixed by brieflyvortexing, and incubated at 30°C for 30 minutes. The transcription reaction was then initiated by adding 3 μL of 10 mM NTP mix and maintained at 30°C for 1 hour. When the transcription reaction was complete, 160 μLstop solution(125 mM Tris-HCl pH 7.5, 12.5 mM EDTA, 150 mM NaCl, 1% SDS) containing 5 μL proteinase K and its reaction buffer was added to the reaction mixture and incubated at 55°C for 30 min. For *in vitro* transcription reactions that included a mutant TFIIB derivative we used HeLa nuclear extract that had the endogenous TFIIB depleted by immunoaffinitychromatography. 10 ngof wild type or mutant derivative TFIIB was added to the reaction system before transcription initiation.

### Acidic phenolpreparation and DNA template elimination

Tris.Cl-balanced phenol (pH 8.0) was used to prepare acidic phenol. In brief, the Tris.Cl-balanced phenol was washed twice by excessive ddH_2_O. The washed phenol was then balanced with an equal volume of 0.1 M HCl buffer on a rocker for 4 hours, the supernatant was changed and pH value was measured 2–3 times during the process until the final pH value of the phenol was below 3.0.

Following digestion with proteinase K, the terminated transcription reaction mixture was subjected to DNA template depletion, namely, extracted by Trizol and acidic phenol on ice for 20 minutes respectively, followed by precipitation with an equal volume of isopropanol. The dried pellet was then dissolved in 200 μLDNase I digestion solution (10μLDNase I, 20 μLreaction buffer and 170 μL ddH_2_O). The DNA template within the transcription reaction mixture was further removed through DNase I digestion at 37°C for 1 hour followed by precipitation with 3 volumes of 100% ethanol.

### Primer extension

Primer extension was performed according to the standard protocol [8] except that the primer used here was specially designed. The primer sequence is as follows: 5’-GTGAAGGTGAAGGTGATGCGgcgtatctcttcatagccttatgc-3’, which comprises sequence from the luciferase gene and GFP gene (The capital letters as shown in the primer). Before the initiation of primer extension, the hybridization reaction between the primer and transcriptswas performed at 42°C for 16 hours.

### Quantitative real time PCR

**Fig 1:**
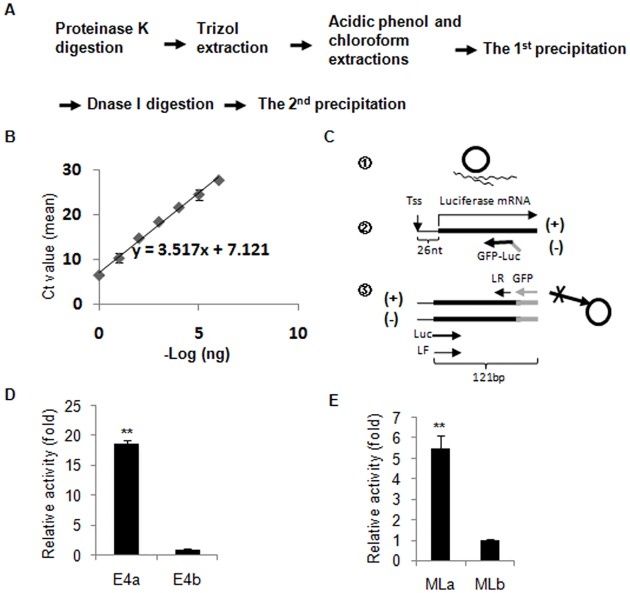
A novel *in vitro* transcription assay using DNA template depletion, primer extension and qPCR. (A) Schematic of the approach used for the DNA template depletion. (B) A standard curve plotted with average Ct value against—log of DNA template quantity (ng). The standard curve was generated by using DNA constructs containing the luciferase gene (pGL3-E4) and qPCR performed with reporter gene primers(LF/LR), the standard curve was used to determine the quantity of DNA template after depletion. (C) A scheme for detecting *in vitro* transcription products using specially designed primers; ① represents the transcription products after the DNA template depletion, which includes the remaining DNA template (circle) and RNA transcript (wave line), ② represents primer extension using a GFP oligonucleotide-tagged luciferase gene reverse primer, in which the GFP oligonucleotide (grey line) linked with the luciferase oligonucleotide ((-))is complementary to luciferase gene RNA transcript (the black straight line and (+)). ③ represents qPCR using cDNA template and the luciferase gene forward primer (black arrow) and GFP gene reverse primer (grey arrow); (-) represents the cDNA template derived from the primer extension, (+) represents the DNA template from the first amplification of the cDNA. LF/LR: Luciferase gene primer pair. (D) Transcription analyses of the E4 promoter under conditions with (E4a) or without the activator GAL4-AH (E4b). (E) Transcription analysis of the ML promoter with (MLa) or without the activator GAL4-AH (MLb). Each bar represents the mean of at least three independent experiments with standard deviation, the symbol “* *” represents P≤0.01, the p values were obtained by performing *t* test.

Quantitative real time PCR (qPCR) was performed with the Bio-Rad SYBR Green reagent and Real Time PCR Detection System. The primers used for qPCR were specially designed to recognize the primer extension-derived product, in which the forward primer is complementary to the 3’ prime sequence of luciferase gene negative strand, which is immediately downstream of the core promoter; the reverse primer is a GFP oligonucleotide that is contained in the primer used for primer extension, the primer pair give rise to a 121bp product. The primer sequences were as follows: forward, 5’-atggaagacgccaaaaacataa-3’(Luc/L);reverse, 5’-gtgaaggtgaaggtgatgcg-3’(GFP/G). In addition, we designed a primer pair for directly detecting luciferase RNA transcript by RT-qPCR or detecting DNA template by qPCR as described in Fig 1C, the primers were as follows: forward, 5’-gaaaggcccggcgccattct-3’(LF); reverse, 5’-ttcatagcttctgccaaccg-3’(LR). The real time PCR data were analyzed by Bio-Rad CFX Manager Software 3.1.

## Results and Discussion

### DNA template elimination

In conventional primer extension-dependent *in vitro* transcription assay, the radioactive isotope-labeled primer is used to detect RNA transcripts that indirectly reflect the efficiency of transcription *in vitro*. To achieve direct detection for transcription product *in vitro* using RT-qPCR, apparently, the DNA template used for transcription must be depleted following the transcription reaction. We first performed an *in vitro* transcription assay using the Adenovirus E4 promoter in the absence or presence of the activator GAL4-AH. When the transcription reaction was completed, the reaction mixture sequentially underwent proteinase K digestion, DNase I digestion, Trizol extraction and precipitation with isopropanol; the pellet was dissolved in 20 μL ddH_2_O, 4 μL of which was used for reverse transcription and detected by qPCR using the reporter gene (luciferase) primers. Unexpectedly, our qPCR data were not consistent with the results as described previously [5], in which the activity of activated transcription for the E4 promoter is significantly lower than that of the basal transcription (data not shown). The underlying problem is because of incomplete elimination of the DNA template as the difference of Ct values for qPCR between the samples with or without DNA depletion is not significant (data not shown).

It has been proposed that it is necessary to remove 99.999% (i.e. left 1pg out of 100ng) of the DNA template in a transcription reaction to minimize the contribution of the template DNA to the final qPCR reaction [14]. We therefore tried to use a more rigorous approach to remove the DNA template from the transcription reaction system (Fig 1a). It has been suggested that acidic phenol can effectively remove DNA contamination [15], thus, in the new approach we added a step to include acidic phenol extraction with prolonged time on ice and a precipitation step before DNase I digestion (Fig 1a). To verify the efficacy of this method, we initially mimicked the procedure of the *in vitro* transcription assay by adding pGL3 basic-E4 template but excluded addition of NTP, and the remaining DNA template within the ‘reaction’ system was detected using reporter gene primers (LF/LR) and qPCR. In the meantime, a standard curve was generated by qPCR using the same DNA template and reporter primers as described above (Fig 1b). As shown in Table 1, the Ct value for E4 qPCRincreased from 5.9 (E4 ctrl1) to 14.9 (E4 (1)) after Trizol and acidic phenol extraction and the first precipitation; the DNase I digestion further increased the Ct value to 21.7. In other words, the level of DNA templatein transcription system dramatically reduced to 0.07 pg from 2.23 ng after a strict DNA template depletion (Table 1). We have achieved similar results in a separate experiment using the AdML DNA template (Table 2). Using these conditions it is now possible to directly detect the products of transcription *in vitro* using both the DNA template depletion and RT-qPCR techniques. However, the results from *in vitro* transcription for E4 promoter by RT-qPCRstill exhibited poor reproducibility whenbasal and activator-dependent transcription were compared (data not shown).

**Table 1:** The Ct values and quantity from qPCR for the E4 reporter vector with or without experiencing the DNA template depletion.

	E4 Ctrl	E4(1)	E4 Ctrl2	E4(2)
Ct(SD)	5.9(0.51)	14.9(0.12)	6.1(0.19)	21.7(0.33)
Quantity(ng,SD)	2.23(0.53)	6.1E-3(4.7E-4)	1.48(0.18)	7E-5(1.5E-5)

E4 ctrl1: E4 reporter vector without Trizol and acidic phenol extraction but including the first precipitation, E4 (1): E4 reporter vector with Trizol and acidic phenol extraction and the first precipitation, E4 ctrl2: E4 reporter vector without Trizol, acidic phenol extraction and DNase I digestion but including the first and second precipitation. E4 (2): E4 reporter vector with Trizol, acidic phenol extraction and DNase I digestion and the first and second precipitation. SD: Standard Deviation.

**Table 2:** The Ct values and quantity from qPCR for the ML reporter vector with or without experiencing the DNA template depletion.

	ML ctrl1	ML (1)	ML ctrl2	ML (2)
Ct(SD)	4.6(0.28)	14.4(0.21)	6.1(0.33)	20.8(0.07)
Quantity(ng,SD)	5.03(0.95)	8.5E-3(1.1E-3)	1.48((0.18)	1.2E-4(6.2E-6)

ML ctrl1: ML reporter vector without Trizol and acidic phenol extraction but including the first precipitation. ML (1): ML reporter vector with Trizol and acidic phenol extraction and the first precipitation. ML ctrl2: ML reporter vector without Trizol, acidic phenol extraction and DNase I digestion but including the first and second precipitation. ML (2): ML reporter vector with Trizol, acidic phenol extraction and DNase I digestion and the first and second precipitation. SD: Standard Deviation.

### A novel method establishment

We supposed that the reason for poor reproducibility in the qPCR-detection of transcripts is likely due to the extreme low efficiency of transcription *in vitro* such that even the very low level of contaminating DNA template could still interfere with qPCR. To solve this problem, instead of directly detecting theRNA transcript by RT-qPCRwe incorporated a prior step of primer extension. We designed the primer used for primer extension to contain an oligonucleotide fragment complementary to the RNA transcript and an oligonucleotide fragment from the GFP gene (GFP-Luc primer). The primers for the subsequent qPCR include a forward luciferase oligonucleotide (Luc or L) and a reverse GFP oligonucleotide (GFP or G). The primers for qPCRwould therefore specifically recognize the cDNAproduced from the primer extension (Fig 1C) as the GFP oligonucletide is absent in the DNA template. To examine the effect of these primers on primer extension and qPCR, we performed primer extension-dependent *in vitro* transcription analysis using E4 and ML reporter vectors incorporating both the DNA template depletion and qPCR techniques. After hybridization, the samples were subjected to primer extension reaction by reverse transcriptase (RT) or placed in a mock reaction that lacked RT. As shown in Table 3, under the conditions without RT, the Ct values for E4 and ML qPCR increased 15 and 13.9 cycles respectively when the ‘reaction’ products were detected by luciferase gene primer pair (LF/LR) and luciferase versus GFP primer pair (L/G). The Ct values for E4 and ML that had a reverse transcription (RT L/G) lied between No RT LF/LRand No RT L/G (Table 3). Taken together, these data confirm that the luciferase versus GFP primer pair (L/G) can indeed specifically recognize the cDNA product from primer extension. However, the luciferase gene primer pair (LF/LR) were not suitable for detecting cDNA from primer extension due to the interference from remnants of the DNA template and the low efficiency of transcription *in vitro*.

**Table 3:** The Ct values from qPCR for E4 and ML *in vitro* transcription assays by primer extension under the conditions with or without reverse transcriptase.

	No RT LF/LR	No RT L/G	RT L/G
E4 Ct(SD)	21.8(0.46)	36.8(0.14)	28.6(0.21)
ML Ct(SD)	23.5(0.86)	37.4(0.45)	28.1(0.06)

No RT LF/LR: *In vitro* transcription assay with mock primer extension but without reverse transcriptase, then detected by qPCR using luciferase gene primer pair (LF/LR). No RT L/G: *In vitro* transcription assay with mock primer extension but without reverse transcriptase, then detected by qPCR using luciferase versus GFP gene primer pair (L/G). RT L/G: *In vitro* transcription assay including primer extension and with reverse transcriptase then detected by qPCR using luciferase versus GFP gene primer pair (L/G). SD: Standard Deviation.

We next examined whether these primers could be used for specifically detecting E4 transcriptsproduced under basal transcription conditions and also in the presence of the transcriptional activator GAL4-AH. Fig 1d illustrates that the activity of the E4 promoter was 19foldhigher in the presence of GAL4-AH than that observed of basal transcription;usingthe conventional detection methodwe previously observed that theactivity of E4 promoter wasenhanced about 10fold by the same activator, demonstrating that the data from this novel method is consistent with our previous results [5]. In a parallel experiment using an ML promoter reporter vector, we have also reproduced the results as described previously [6, 16] (Fig 1e). Together, these data suggest that our novel nonradioactive method is capable of detecting the *in vitro* transcription activity of either E4 or ML promoter-directed reporter gene in both the absence and presence of a transcriptional activator. This method is based on the property that the GFP oligonucleotide-tagged cDNA is specifically recognized by the primers used for qPCR, in which the GFP reverse primer cannot bind to the remaining DNA template within the transcription system after the DNA template depletion (Fig 1c). Although the luciferase forward primer can bind to DNA template, its extended product during PCR is null for the GFP reverse primer. Another important factor is that the concentration of the primer used in the primer extension is usually less than 0.05 μM, which is over 100 times lower than that of the primers used for qPCR (6.25 μM). Therefore, the primer used for primer extension has very little opportunity to generate another source of DNA template during qPCR after DNA template elimination although it is possible that it can bind to the original DNA template. Nevertheless, our data show that the samples that did not contain RT still produce some background by qPCR using the luciferase versus GFP primer pair (L/G), but the Ct values lagged 8–9 cycles when compared to the samples with RT (Table 3). This level of background does not significantly impact the accuracy of qPCR for the sample that contains RT because the qPCRstep only counts the cycles of threshold, i.e. the cycle number when the fluorescence is at the beginning of exponential growth. This residual background can be solved by diluting the concentration of the sample or by shortening the thermal cycle number for qPCR.

### Method validation

**Fig 2:**
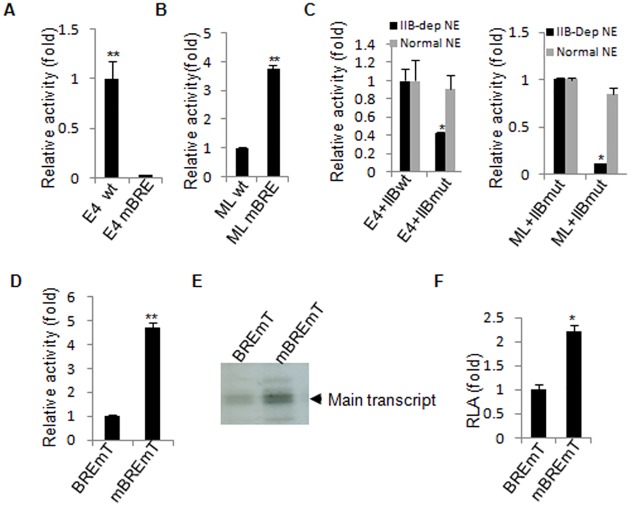
Validation of the novel method by analysis of core promoter elements. A) The analysis of basalactivator-independent transcription of the wild type E4 promoter and an E4 derivative that contains a defective BRE. B) The analysis of basal activator-independent transcription of the wild type ML promoter and an ML derivative that contains a defective BRE. C) The effect of TFIIB mutation (G153Q:R154A) on the activity of basal transcription for the E4 promoter (left panel) and ML promoter (right panel) using TFIIB-depleted and non—depleted NE supplemented with wild type TFIIB or its mutant. D) Analysis of transcription activation usingthe promoters BRE-mTATA (BREmT) and mBRE-mTATA (mBREmT), using nonradioactive *in vitro* transcription assay. E) As in part D but using a conventional *in vitro* transcription assay followed by electrophoresis and detection by autoradiography. F) The AdML promoter derivatives BRE-mTATA (BREmT) and mBRE-mTATA (mBREmT)linked to a luciferase reporter were co-transfected with a vector driving expression of the activator GAL4-VP16. 48 hours later the cells were lysed and luciferase activity was quantified. Each bar represents the mean of at least three independent experiments with standard deviation. The symbol “*” represents P≤0.05, the symbol “**” represents P≤0.01,the p values were obtained by performing *t t*est.

Besides analysis of basal and activator-dependent transcription, the Adenovirus E4 and ML core promoters are often used for the studyof corepromoter element and transcription factor function [5–6, 16]. To validate whether the method is suitable for such analyses, we have generated E4 and ML promoterreporter vectors that contains a defective BRE (E4mBRE and MLmBRE). We performed *in vitro* transcription analysis alongsidewild typeE4 and ML promoter reporters under basaland activator-independent conditions using the established method. The transcription activity of the E4 mBREwas reduced over 30-fold when compared to that of the wild type E4 promoter, whereas the transcription activity of the ML mBRE derivative increased 3.7 fold when compared to that of the wild type ML promoter (Fig 2a and 2b). Our previous work showed that the activity of E4 promoter wasreduced 30% whenthe BRE was rendered defective [5], whereas the activity of the AdML promoter was increased 3-fold by the defective BRE [6,16]. Our data further demonstrate that the results obtained by the new methodpresented herewere consistent with thosefrom the conventional in vitro transcription assay. We reported that a loop in the first direct repeat of TFIIB makes sequence-specific contact with BRE downstream of the TATA box (BRE^d^) at E4 promoter. The TFIIB mutant derivative (G153Q:R154A) is defective in interaction with theBRE^d^ and exhibits decreased transcription activity at the E4 promoter [5]. Therefore, we performed basal (activator-independent)transcription analysis using theE4 and ML promoters using TFIIB-depleted and non-depleted nuclear extract supplemented with purified recombinant wild type TFIIB or TFIIB mutant (G153Q:R154A). Fig 2C shows that TFIIB mutant exhibited significantly reduced activity using either the E4or ML promoter, TFIIB mutant had little impact on transcription of the E4 and AdML promoters using the NE that endogenous TFIIB was not depleted. Taken together, the data confirm that our newly established method can be applied to several types of transcriptional analyses *in vitro*.

We previously demonstrated and show again above (Fig 2B)that mutation of the BRE stimulates the activity of the AdML promoter [6]. Whether this effectdepends on the TATA box of the AdML promoter remains unknown. We next examined the effect of BRE mutation on the transcription activity of the AdML promoter when the TATA element was also rendered defective. The transcriptional activity of the AdMLpromoter containing a defective TATA element (BREmTATA) was compared with the AdML promoter containing both a defective TATA element and defective BRE (mBREmTATA) using both nonradioactive (Fig 2D) and conventional (Fig 2E)*in vitro* transcription methods. Mutation of the BRE in the AdML promoter that also has a defective TATA element significantlyincreasedtranscription of the TATA-less promoter. Taken together with our previous work showing that the BRE acts as a negative element in the context of a wild type TATA element [6,16], this suggests that BRE function for AdML promoter is independent of the TATA box. The data from luciferase assays comparing BREmTATA with mBREmTATA further confirm the finding (Fig 2F). Since three parallel methods lead to a consistent observation (Fig 2D, 2E and 2F),it indicates that the nonradioactive method for *in vitro* transcription can substitute for the conventional method.

The conventional primer extension-dependent *in vitro* transcription assay is a popular approach in the research field of gene transcription because of its strong sensitivity and because it directly detects RNA product by using radioactive isotope-labeled primer, however, its drawbacks are also notable. In this study, we have established a novel nonradioactive method for *in vitro* transcription analysis using DNA template elimination, primer extension and qPCR techniques. Recently, Park and Magan [14] reported a method for cell-free *in vitro* transcription that can directly detect RNA transcripts by DNA template depletion and one-step RT-qPCR. In this method they used a promoter-linked G-less cassette as DNA template and immobilized beads to remove the DNA template after the transcription reaction was completed. We initially sought to use a similar method to analyze transcription of the E4 reporter vector; however, we were not able to achieve stable results, although the DNA template in our experiment was depleted to a similar level [14]; it is likely that this is due to the low efficiency of transcription for E4 and ML reporter vectors. Voss *et al* [17] have recently reported another approach that can directly detect RNA transcripts *in vitro* using AffimetricsQuantigene techniques, but it relies on specialist and facilities to complete the experiment. In our study, the uniquely designed primers have been used to solve the problem; our data indicate that the primers successfully detect low levels of RNA transcript by primer extension and qPCR. Our novel method is distinct from those published recently [14, 17] and displays high sensitivity, simplicity and low cost. The method will therefore benefit numerous laboratories that need *in vitro* transcription analysis but cannot easily employ approaches that use radioactive isotopes.

## References

[1] Fan H, Sugiura M. Basal and activated in vitro transcription in plant by RNA polymerase II and III. MethodsEnzymol. 1996;273 (Part A): 268–77. 10.1016/s0076-6879(96)73025-2 8791618

[2] Lagrange T, Kapanidis AN, Tang H, Reinberg D, Ebright RH. New core promoter element in RNA polymerase II-dependent transcription: sequence-specific DNA binding by transcriptionfactorIIB.Genes Dev. 1998 1 1; 12: 34–44. 9420329 10.1101/gad.12.1.34 PMC316406

[3] Willy PJ, Kobayashi R, Kadonaga JT. A basal transcription factor that activates or represses transcription.Science 2000 11 3; 290: 982–95. 11062130 10.1126/science.290.5493.982

[4] Stepanchick A, Zhi H, Cavanaugh AH, Rothblum K, Schneider DA, Rothblum LI. DNA binding by the ribosomal DNA transcription factor rrn3 is essential for ribosomal DNA transcription. J. Biol. Chem. 2013 3 29; 288: 9135–44. 10.1074/jbc.M112.444265 23393135 PMC3610986

[5] Deng W, Roberts SG. A core promoter element downstream of the TATA box that is recognized by TFIIB. Genes Dev.2005 10 15; 19:2418–23. 16230532 10.1101/gad.342405 PMC1257396

[6] Deng W, Malecová B, Oelgeschläger T, Roberts SG. TFIIB recognition elements control the TFIIA-NC2 axis in transcriptional regulation. Mol. Cell Biol. 2009 3; 29: 1389–00. 10.1128/MCB.01346-08 19114554 PMC2648230

[7] Deng W, Lopez-Camacho C, Tang JY, Mendoza-Villanueva DA, Jackson DA, Shore P. Cytoskeletal protein filamin A is a nucleolar protein that suppresses ribosomal RNA gene transcription.Proc. Natl. Acad. Sci. (U.S.A.) 2012 1 31;109: 1524–29. 22307607 10.1073/pnas.1107879109 PMC3277164

[8] Carey MF, Peterson CL, Smale ST. In vitrotranscription using HeLa cell extracts and primerextension ColdSpringHarb Protoc.2009 12; pdb.prot5331 10.1101/pdb.prot5331 20150078

[9] Bustamante M, Jin J, Casagran O, Nolan T, Riechmann JL. Geneexpressionanalysis by quantitative real-time PCR for floral tissues.Methods Mol. Biol. 2014; 1110: 363–82. 10.1007/978-1-4614-9408-9_21 24395270

[10] Ozalp VC, Bayramoglu G, Kavruk M, Keskin BB, Oktem HA, Arica MY. Pathogendetection by core-shell type aptamer-magnetic preconcentration coupled to real-time PCR.Anal. Biochem.2014 2 15; 447: 119–25. 10.1016/j.ab.2013.11.022 24291643

[11] Ivanova M, Singh R, Dharmasena M, Gong C, Krastanov A, Jiang X. Rapid dentification of Campylobacter jejuni from poultry carcasses and slaughtering environment samples by real-time PCR.Poult. Sci. 2014 6; 93: 1587–97. 10.3382/ps.2013-03736 24879709

[12] Ozkaya-Parlakay A, Cengiz AB, Ceyhan M, Hascelik G, Kara A, Celik M, et al Evaluation of multiplex real time polymerase chain reaction and procalcitonin in the diagnosis of sepsis.Clin. Lab.2014; 60: 1075–81. 25134374 10.7754/clin.lab.2013.130732

[13] Dignam JD, Lebovitz RM, Roeder RG. Accurate transcription initiation by RNA polymerase II in a soluble extract from isolated mammalian nuclei. Nucleic Acids Res. 1983 3 11; 11: 1475–9. 6828386 10.1093/nar/11.5.1475 PMC325809

[14] Park JH, Magan N. Reverse transcriptase-coupled quantitative real time PCR analysis of cell-free transcription on the chromatin-assembled p21 promoter. PLOS One 2011; 6(8): e23617 10.1371/journal.pone.0023617 21886803 PMC3160311

[15] Siebert PD, Chenchik A. Modified acid guanidiniumthiocyanate-phenol-chloroform RNA extraction method which greatly reduces DNA contamination. Nucleic Acids Res. 1993 4 25; 21: 2019–20 7684133 10.1093/nar/21.8.2019 PMC309453

[16] Evans R, Fairley JA, Roberts SG. Activator-mediated disruption of sequence-specific DNA contacts by the general transcription factor TFIIB. Genes Dev. 2001 11 15; 15(22): 2945–49. 11711430 10.1101/gad.206901 PMC312826

[17] Voss C, Schmitt B, Werner-Simon S, Lutz C, Simon W, Anderl J. A novel, non-radioactiveeukaryoticin vitro transcription assay for sensitive quantification of RNA polymerase II activity. BMC Mol. Biol. 2014 4 3; 15:7 10.1186/1471-2199-15-7 24694320 PMC4021065

